# Positive- and negative-acting regulatory elements contribute to the tissue-specific expression of *INNER NO OUTER*, a YABBY-type transcription factor gene in Arabidopsis

**DOI:** 10.1186/1471-2229-12-214

**Published:** 2012-11-13

**Authors:** Marissa K Simon, Luis A Williams, Kristina Brady-Passerini, Ryan H Brown, Charles S Gasser

**Affiliations:** 1 Department of Molecular and Cellular Biology, University of California, Davis, CA 95616, USA; 2 HHMI, Harvard University, Cambridge, MA 02138, USA; 3 General Mills, Kannapolis, NC 28081, USA

**Keywords:** Promoter, Enhancer, Ovule, Brassica, Plant development

## Abstract

**Background:**

The *INNER NO OUTER* (*INO*) gene, which encodes a YABBY-type transcription factor, specifies and promotes the growth of the outer integument of the ovule in Arabidopsis. *INO* expression is limited to the abaxial cell layer of the developing outer integument of the ovule and is regulated by multiple regions of the *INO* promoter, including POS9, a positive element that when present in quadruplicate can produce low-level expression in the normal *INO* pattern.

**Results:**

Significant redundancy in activity between different regions of the *INO* promoter is demonstrated. For specific regulatory elements, multimerization or the addition of the cauliflower mosaic virus 35S general enhancer was able to activate expression of reporter gene constructs that were otherwise incapable of expression on their own. A new promoter element, POS6, is defined and is shown to include sufficient positive regulatory information to reproduce the endogenous pattern of expression in ovules, but other promoter regions are necessary to fully suppress expression outside of ovules. The full-length *INO* promoter, but not any of the *INO* promoter deletions tested, is able to act as an enhancer-blocking insulator to prevent the ectopic activation of expression by the 35S enhancer. Sequence conservation between the promoter regions of *Arabidopsis thaliana*, *Brassica oleracea* and *Brassica rapa* aligns closely with the functional definition of the POS6 and POS9 regions, and with a defined *INO* minimal promoter. The *B*. *oleracea INO* promoter is sufficient to promote a similar pattern and level of reporter gene expression in Arabidopsis to that observed for the Arabidopsis promoter.

**Conclusions:**

At least two independent regions of the *INO* promoter contain sufficient regulatory information to direct the specific pattern but not the level of *INO* gene expression. These regulatory regions act in a partially redundant manner to promote the expression in a specific pattern in the ovule and suppress expression outside of ovules. Establishment of this pattern requires cooperation and competition between multiple positive and negative regulatory elements.

## Background

Specific spatiotemporal patterns of gene expression are required for some developmental processes and may depend on the action of both positive and negative factors for transcriptional regulation. Sequence-specific DNA-binding transcription factors act in a combinatorial manner on multiple binding sites in DNA to achieve different transcriptional outputs [1]. This type of combinatorial control is a major mechanism underlying eukaryotic transcriptional regulation and is responsible for differential gene expression [2,3]. For example, the *SPATULA* (*SPT*) gene in Arabidopsis is specifically expressed in multiple tissues and the *SPT* promoter includes multiple tissue-specific enhancers and silencers, and shows several regions of conservation with the promoters of orthologous genes in closely related species [4]. The putative promoter region of *FILAMENTOUS FLOWER* (*FIL*), a YABBY gene family member, has also been characterized and shown to include both negatively and positively acting regions that together cause it to be expressed in the abaxial regions of primary lateral organs [5]. *INNER NO OUTER* (*INO*) is another Arabidopsis YABBY gene that is expressed in abaxial tissues, but exclusively in the outer integument of ovules [6,7]. To understand the combinatorial role of multiple regulatory regions to produce the highly specific pattern of *INO* expression we have investigated promoter regions of this gene in Arabidopsis.

The *INO* gene promotes the initiation and growth of the outer integument on the gynobasal side of the ovule to produce an amphitropous (recurved) shape [7-10]. *INO* is expressed at the initiation site and in the developing abaxial layer of the outer integument in ovules [6,7,11,12]. *SUPERMAN* (*SUP*) acts to limit the growth of the outer integument to the gynobasal side of the ovule, and *sup* mutants show overproliferation of the outer integument on the gynoapical side of the ovule resulting in a more orthotropous ovule [10]. The maintenance and up-regulation, but not the initiation, of *INO* expression requires active INO protein, and SUP suppresses the autoregulatory action of INO [11,13].

Reporter gene and complementation analyses have previously identified a 2.3 kb region upstream of the INO coding sequence to contain regulatory information sufficient for correct *INO* expression, termed P-INO [11]. Deletion experiments using ß-glucuronidase (GUS) enzymatic activity as a reporter defined a 295 bp positive regulatory element within P-INO, which was termed POS9 [12]. While a single copy of POS9 did not produce detectable expression, when this element was present in combination with either the 5^′^ (termed POSX) or 3^′^ (termed POSY) P-INO flanking regions, or at least three additional copies of POS9, the wild-type pattern of INO expression was reproduced. This indicated the presence of additional, at least partially redundant, positive regulatory elements in P-INO [12]. No elements with negative regulatory activity were identified in this study.

The Meister et al. [12] study showed that POS9 included information that was sufficient to produce the normal pattern of expression and also contributed to the quantitative level of expression. Redundant quantitative information was also demonstrated for the regions 5^′^ and 3^′^ of POS9, but whether these regions also included redundant positional information was not clear from these prior studies. We have now utilized the reporter gene methods to test for the presence of such information. We have also used the enhancer region of the cauliflower mosaic virus 35S transcript promoter to see if such a general enhancer can substitute for the P-INO regions providing quantitative expression in the ovule. Evaluation of sequence conservation in other members of the Brassicaceae has allowed us to focus our efforts on the most conserved regions of the promoter. In these studies we find redundancy in both quantitative and positional activities among the different regions of P-INO, and further find evidence of negative regulatory activity in elements of P-INO. Attempts to identify specific functional sequence motifs within the promoter were unsuccessful.

## Results

**Figure 1:**
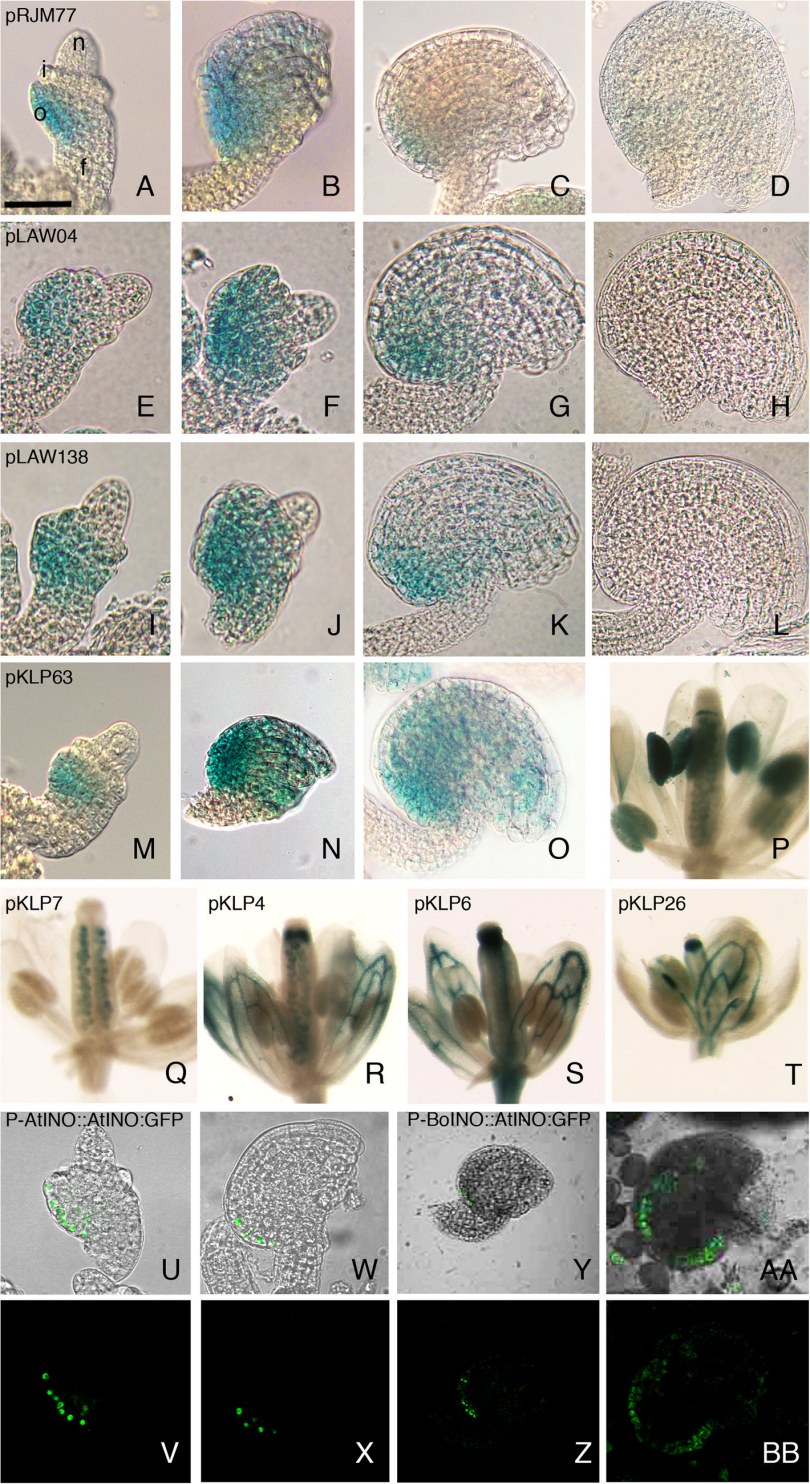
**Expression patterns of P-INO promoter constructs in wild-type plants.** DIC (**AO**) and light microscopy images of ovules and flowers from plants containing pRJM77 (P-INO::GUS) (**AD**), pLAW04 (POSX 3′delPOSY to −756::GUS) (**EH**), pLAW138 (POSX 5′delPOSY to −868::GUS) (**IL**), pKLP63 (4XPOS6::GUS) (**MP**), pKLP7 (35Senh:P-INO::GUS) (**Q**), pKLP4 (35Senh:POSXPOSY::GUS) (**R**), pKLP6 (35Senh:POSX::GUS) (**S**) and pKLP26 (35Senh::35SMP::GUS) (**T**), assayed for GUS activity. Panels (**IL**) were stained for GUS activity using a 50X elevated concentration of 5-bromo-4-chloro-3-indolyl β-d-glucuronic acid (X-Gluc) relative to panels (**AH** and **MT**), to detect GUS activity with intensity and pattern similar to that observed for the full-length promoter. Stages of ovule development: 2-III (A, I, M); 2-IV (E); 2-V (F, J, U); 3-I (B, N, W); 3-III (C, G, K, Y); 4-III (D, H, L, O, AA) (stages according to Schneitz et al. [14]). Confocal microscopy with the constructs P-AtINO::AtINO:GFP [11] (U-X) and P-BoINO::AtINO:GFP (Y-BB), in Arabidopsis. (U, W, Y and AA) show a DIC image overlaid with the green confocal signal from the GFP, or (V, X, Z and BB) show GFP florescence alone. Scale bar: (F) 15 μm, (A, B, E, I, J, M, U, V) 25 μm, (G, N, W, X) 30 μm, (C, K, O) 40 μm, (D, H, L, Y, Z) 50 μm, (P, Q, R, S, T, AA, BB) 700 μm. f, funiculus; o, outer integument; i, inner integument; n, nucellus.

To further dissect the *INO* promoter, various promoter constructs were evaluated for their ability to replicate the pattern of expression produced by the full-length promoter using the GUS reporter gene. *INO* expression pattern, as revealed by GUS staining in ovules of P-INO::GUS transformants, has been previously described (Figure 1A-D) [11,12]. GUS activity was first detected in the outer integument primordium at stage 2-III (stages according to Schneitz et al. [14]), it remained restricted to the outer integument and persisted through development of this structure until after stage 3-I. The current reporter gene analyses were initially carried out using the concentration of substrate used in the earlier study [11], but for some of the promoter constructs staining with an elevated substrate concentration was also evaluated.

### Conservation of INO promoter within Brassicaceae

Significant conservation in the pattern of *INO* expression has been observed in the small number of additional angiosperm species in which expression of *INO* orthologs has been examined [15-17]. To help identify important regions of P-INO the conservation and divergence of promoter regions was examined in *INO* orthologs of *Brassica oleracea* and *Brassica rapa* (which has two homeologous *INO* genes), members of Brassicaceae with ovule morphology similar to that of Arabidopsis.

**Figure 2:**
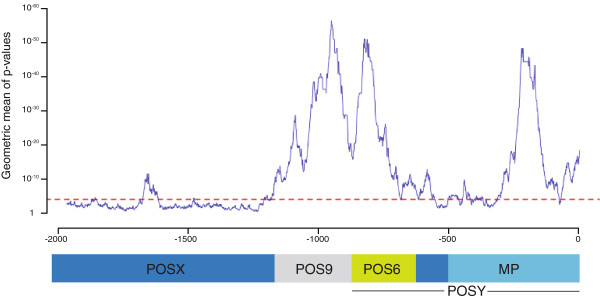
**Multi-species conservation profile for the *INO *promoters. ***INO* promoters from *Arabidopsis thaliana*, *Brassica oleracea* and *Brassica rapa* orthologs were compared utilizing the EARS tool using a 60-bp window [18]. The dashed line indicates the significance threshold (P=0.0001). The diagram at bottom shows locations of the functional regulatory domains identified through deletion analyses in the Arabidopsis *INO* promoter. MP, minimal promoter.

Three alignment-based methods were used to compare the 5^′^-flanking regions of *AtINO*, *BoINO* and the two *BrINO* genes to identify conservation profiles in the putative *INO* promoters. The profiles identified several regions of extended conservation along the *INO* promoters. Three regions of most significant conservation were identified by the EARS (Evolutionary Analysis of Regulatory Sequences) [18] method and these were confirmed by either or both of the Clustal and FSA (Fast Sequence Alignment) procedures [18-20] (Figure 2). The alignments also revealed multiple short regions of higher similarity (Additional file 1). A 54 bp region with 74% identity extends from position −1711 to −1657, and is the only highly similar element located upstream of previously identified POS9 positive regulatory element. Two regions were identified with high similarity within the POS9 region, -1158 to −1090 and −1066 to −898, with 84% identity in each region. An additional region, from −869 to −774 with 85% identity, overlaps the 3′terminus of the POS9 element and additional downstream sequence. A final segment from −317 to −173 exhibits 77% sequence identity. These values compare with the overall 37% identity among the four *INO* promoters examined (FSA method, [20]).

### *Brassica* INO promoter activity in Arabidopsis

To test the transcriptional activity of the *B*. *oleracea INO* promoter (P-BoINO) in Arabidopsis, constructs were made with P-BoINO and the coding sequences of At*INO*, ß-glucuronidase, and green fluorescent protein (GFP).

A reporter construct, P-BoINO::AtINO:GFP, that included 2.4 kb of 5^′^ flanking sequence of *BoINO* including the highly conserved sequences, was transformed into Arabidopsis. The transgenic plants showed GFP fluorescence on the gynobasal side of the developing ovules in the outer layer of the outer integument, that mirrored the results seen with P-AtINO::AtINO:GFP (Figure 1U-BB). Similar results were obtained for expression of the P-BoINO::GUS construct (data not shown).

The GFP and GUS data indicated that P-BoINO can qualitatively promote a similar pattern of expression to P-AtINO when in the presence of a normal endogenous *INO* gene. The *INO* gene has been shown to amplify expression from its own promoter in an autoregulatory loop [11]. To test if P-BoINO could also participate in the autoregulatory loop, we tested the ability of a P-BoINO::AtINO transgene to complement the *ino1* mutation. Five of six homozygous *ino1* plants transformed with the P-BoINO::AtINO construct exhibited restoration of female fertility and produced ovules resembling those of wild-type (data not shown), indicating complementation by the construct.

### Deletion analyses delineate a complex proximal promoter element with positive and negative acting regions

A previous study showed that the POS9 element was able to replicate the endogenous expression pattern only when in combination with additional copies of POS9, or with either the region of P-INO 5^′^ or the region of P-INO 3^′^ of POS9 [12]. This suggested the presence of additional positive elements within the 5^′^- and 3^′^-regions, and these regions were designated POSX and POSY, respectively [12]. The presence of important functional information in the POSY region was further suggested by sequence conservation in this region in *Brassica* species (Figure 2, Additional Figure 1).

To define the functional regions in the POSY element, a combination of PCR and oligonucleotide-mediated mutagenesis [21] was used to generate five POSY 3^′^-terminus and eight POSY 5^′^-terminus deletions, in a plasmid already lacking POSX. A minimal promoter element from the cauliflower mosaic virus (CaMV) 35S promoter, previously shown to function in transcription initiation in these type of analyses [12], was used to compensate for the removal of endogenous minimal elements required for transcription initation in the deletion constructs. GUS activity was assayed in a minimum of ten primary transformants for each construct.

**Figure 3:**
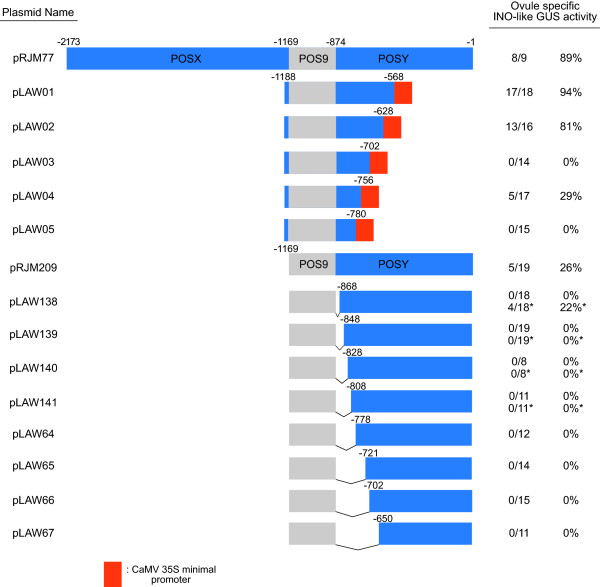
**Ovule expression from combinations of POS9 and truncated forms of POSY.** Diagrams of the entire *INO* promoter and POSY deletion constructs are shown at left. Positions in the diagrams represent the number of base pairs upstream of the putative *INO* translational start codon. The orange box represents a 60 bp minimal promoter element from the CaMV 35S promoter. Ovule specific *INO*-like GUS activity indicates the number of independent plants exhibiting the outer integument specific GUS staining pattern shown by the full-length P-INO relative to the total number of transformants evaluated, and percentage calculated from these values. The frequency of *INO*-like of expression for pRJM77 was previously described [12], and was similar to that obtained in the experimental repetitions reported here. *Results obtained when a 50X elevated concentration of X-gluc substrate was used.

Deletion from the 3′-end of POSY to position −628 (pLAW02) did not compromise the activity of the reporter gene, as thirteen of sixteen transformants showed GUS staining in ovules in a pattern comparable to that generated by the full length promoter (wild-type expression pattern) (Figure 3). While deletion to position −702 (pLAW03) abolished GUS activity, removal of an additional 54 bp to position −756 (pLAW04) restored wild-type GUS expression pattern in five of seventeen transformants assayed (Figure 1E-H). This indicated the presence of a negative regulatory element in the 54 bp region between −756 and −702. Further deletion to position −780 (pLAW05) led to failure to produce detectable GUS activity in any of the fifteen transformants tested indicating the presence of important positive regulatory information in this region between −780 and −756.

Deletion of only 6 bp (pLAW138, deletion to −868) from the 5′-end of the POSY region led to a loss of staining under our standard conditions, and this was also true of all further 5′-deletions (POS95′POSYdel::GUS constructs) of this region (Figure 3). Transgenic lines with the smallest deletions, pLAW138 (deletion to −868), pLAW139 (−848), pLAW140 (−828) and pLAW141 (−808)) were also assayed using 10X increased histochemical substrate concentration but did not show any visible GUS activity (Figure 3 and data not shown). When substrate was elevated to 50X, GUS wild-type staining pattern was detected in four of eighteen pLAW138 transformants, but no staining was visible in lines containing any of the larger deletions (Figures 1I-L and 3). Since even a deletion of only 6 bp at the extreme 5^′^ -end of the region containing POSY dramatically affected expression, these observations indicate the presence of sequence information at the 5′-terminus of POSY that is relevant for correct levels of expression in the absence of POSX. Taken together, these analyses of P-INO deletion constructs define a 246 bp element within the POSY region that we now designate POS6 for its position approximately 600 bp upstream of the INO start codon. The nucleotide immediately downstream of POS9 is considered the 5′-terminus of POS6, while its 3′-terminus was positioned 628 bp upstream of the putative INO translational start codon. The borders of POS6 correlate with the region of significant conservation with promoter regions of *INO* orthologs in *Brassica* species (Figure 2).

### Redundancy of P-INO positive regulatory elements

**Figure 4:**
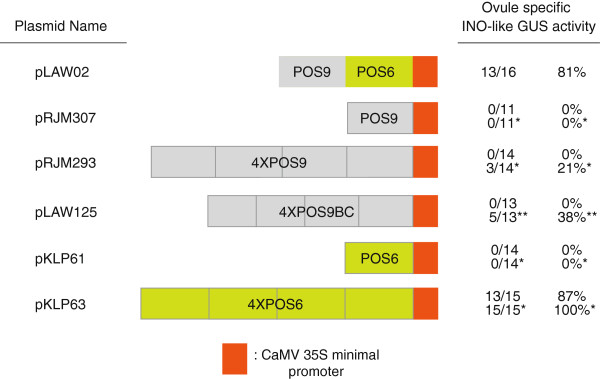
**Multiple copies of POS9 and POS6 regions can replicate P-INO function.** Structures of the POS9 (−1187 to −874) and POS6 (−873 to −628) promoter region constructs are shown. The orange box represents a 60 bp minimal promoter element from the CaMV 35S promoter. Ovule specific INO-like GUS activity indicates the number of plants exhibiting the outer integument specific GUS staining pattern shown by the full-length P-INO relative to the total number of transformants evaluated, and percentage calculated from these values. * Results obtained when an 8X elevated concentration of X-gluc substrate was used. ** Results obtained when a 5X elevated concentration of X-gluc substrate was used. The frequencies of patterns of expression for pRJM307 and pRJM293 were previously described [12], and were experimentally repeated in this assay with similar results shown here.

When included together with a minimal promoter region, the combination of POS9 and POS6 (pLAW02) was sufficient to replicate expression from the entire P-INO. In contrast, neither POS9 alone nor POS6 alone was able to produce detectable expression from the minimal promoter, even with elevated substrate concentration (Figure 4). However, previous reporter analyses showed the POS9 element to be sufficient to replicate the *INO* expression pattern (but at a lower level of expression) when present in four or more tandem copies [12] (Figure 4). Further dissection of POS9, indicated the presence of at least three different sub-elements, POS9A, POS9B and POS9C [12]. POS9A was proposed to contain at least an enhancer of transcription, since its removal (in the presence of POSY) affected only the levels of early expression of GUS [12]. To further characterize the POS9 element, a tetramer of a 178 bp sub-fragment of POS9 that contained only POS9BC, was constructed in conjunction with the 35S minimal promoter (pLAW125, Figure 4). GUS activity was not detected in any of thirteen 4XPOS9BC::GUS transformants assayed at standard conditions. However, when a 50X concentration of substrate was used, five of thirteen transformants exhibited GUS staining in a wild-type *INO* pattern. These results indicate that regulatory information sufficient to replicate the pattern of expression of P-INO is contained within POS9BC, but that the sequences in the POS9A region have a positive effect on transcription levels.

**Figure 5:**
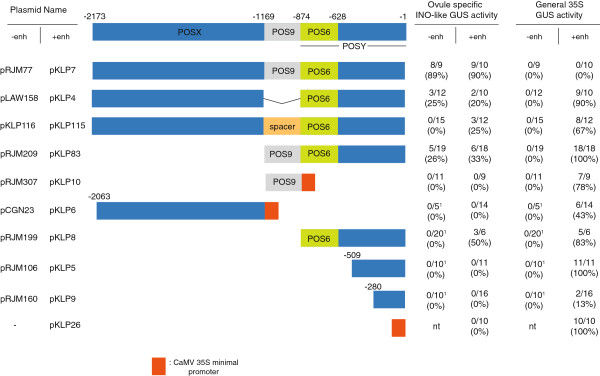
**Effects of the CaMV 35S enhancer on P-INO deletion construct activity.** Diagrams show the entire *INO* promoter and promoter deletion constructs with the first plasmid name designating the original construct, and the second name designating the same construct but with the 35S enhancer added at the extreme 5^′^-end of the construct. pRJM77 is the construct with the entire P-INO [12]. The orange box represents a 60 bp minimal promoter element from the cauliflower mosaic virus (CaMV) 35S promoter. Ovule specific INO-like GUS activity indicates the number of plants exhibiting the outer integument specific GUS staining pattern shown by the full-length P-INO relative to the total number of transformants evaluated (and percentage expressing based on these values), and general 35S GUS activity indicates the number of plants exhibiting a 35S pattern of GUS activity observed in flowers relative to the total number of transformants evaluated. Results are given for constructs without (−enh) and with (+enh) the addition of the 35S enhancer. The frequencies of patterns of expression for pRJM77, pRJM209 and pRJM307 were previously described [12], and were similar to those obtained in the experimental repetitions reported here. ^1^The frequencies of patterns of expression for pCGN23, pRJM199, pRJM106 and pRJM160 are those from the previous report [12]. nt, not tested.

When combined with POS9, the flanking POSX and POSY regions of P-INO also contribute to expression in the ovule. To test if these two regions together would be sufficient to promote the appropriate pattern of GUS expression a POSXPOSY::GUS construct, deleting POS9, was prepared (pLAW158). Three of twelve transformants with this construct showed *INO*-like ovule-specific expression (Figure 5). To test if these regions could produce the same effect in their native spatial relationship, an additional construct was prepared in which a spacer region of the same size and relative GC content as POS9 was introduced between POSX and POSY. The POSXspacerPOSY::GUS showed no expression of the reporter gene, as zero of fifteen transformants stained in the ovule (Figure 5). This indicates that the activity of one or both of these elements has an activity similar to that of POS9, but this activity is most effective when in close proximity to a second positive element.

While a single copy of POS6 (in conjunction with a minimal promoter) was insufficient to produce detectable ovule expression ([12] and Figure 4), four copies of this region in the 4XPOS6::GUS construct (pKLP63) were sufficient to replicate the *INO* expression pattern in the ovules of thirteen out of fifteen transformants using standard staining conditions, and all transformants showed ovule staining at higher substrate concentrations (Figure 1M-O and 4). In addition, two of fifteen transformants showed additional expression of the reporter gene in the anthers when assayed under standard conditions, and fourteen out of fifteen transformants showed this staining when assayed at a higher substrate concentrations (Figure 1P). This specific ectopic pattern of expression is also observed at a lower frequency in 1XPOS6::GUS and 2XPOS6::GUS transformants suggesting that the anther expression is induced by the POS6 element and is not an artifact of the tetramerization of the POS6 element (Figure 4 and data not shown). Staining outside of the ovule was not detected in the 4XPOS9::GUS or the POS9POS6::GUS transformants. Thus, the POS6 region contains positive regulatory information to activate transcription of the reporter gene in an *INO*-like pattern in the ovule and also in other tissues of the plant. These data suggest that the *INO* promoter contains multiple positive regulatory elements sufficient for the proper pattern of *INO* expression, but these elements are not individually sufficient to induce expression to a detectable level. Further, elements within the POS6 region can promote expression in tissues outside of the ovule, but this activity is suppressed by sequences included in POS9 and POSX.

### Activity of the CaMV 35S general enhancer fused to regions of P-INO

The general enhancer of the CaMV 35S promoter [22] was used to further evaluate the functions of the different regions of P-INO. The 35S general enhancer is expected to promote gene expression in a variety of plant structures [23]. We confirmed this activity with a control construct, pKLP26, which fused the 35S enhancer to the 35S minimal promoter and GUS coding region. All ten transformants evaluated showed GUS activity in ovules (but not in the pattern produced by P-INO) and other parts of the plant (Figures 1T and 5).

For some genes, the minimal promoter alone includes sufficient information for tissue-specific regulation [24]. To test if this was the case for *INO*, and to identify the minimal promoter, we fused the 35S enhancer to proximal regions of P-INO. Previously reported 5^′^ deletions to positions −509 (pRJM106) and −280 (pRJM160) failed to produce any reporter gene expression [12]. The addition of the 35S enhancer to the −509 bp deletion of P-INO (pKLP5) did not result in expression in the ovules of any of eleven transformants, but all eleven did exhibit expression in non-ovule regions of the plant (Figure 5). This demonstrates that this region contains sufficient information to act as a minimal promoter, but provides no evidence of inclusion of information for tissue-specific expression. Addition of the 35S enhancer to the −280 bp P-INO deletion (pKLP9) also showed no expression in ovules in any of sixteen transformants, and showed non-ovule expression in only two of sixteen transformants, suggesting that this region may not contain sufficient information to act as an efficient minimal promoter (Figure 5).

The POSX, POS9 and POS6/POSY regions of P-INO contain regulatory elements that promote expression in the abaxial layer of the outer integument, but each of these elements alone was insufficient to promote expression by itself. Expression was only seen when each of these was combined with a second P-INO element ([12] and Figure 5). To test if the contribution of a second element was simple enhancer activity we evaluated the ability of CaMV 35S promoter general enhancer [22] to substitute for a second P-INO region. The separate addition of the 35S enhancer to each of these regions (pKLP6, pKLP10, and pKLP8, respectively) led to expression outside the ovule in nearly all plants for POSY (5 of 6) and POS9 (7 of 9) and for nearly half of plants for POSX (6 of 14) (Figure 1S). In contrast, INO-like expression was observed only for addition of the 35S enhancer to POSY where three of six transformants showed this pattern (Figure 5). Thus, expression from POSX and POS9 in ovules may require additional tissue-specific positive elements and activation by a general enhancer was unable to activate such expression from these regions.

Some pairwise combinations of P-INO elements produce expression in the correct spatial pattern, but at a lower level (or lower frequency) than the full P-INO promoter. We tested the effects of addition of the 35S enhancer on such pairwise combinations of the P-INO elements. Addition of the enhancer to POS9POSY, or POSXPOSY combinations (pKLP83 and pKLP4, respectively) did not lead to significant change in the frequency of detectable INO-like expression in the ovule, and consistently led to expression outside of ovules (Figure 1R and 5).

The 35S enhancer was also fused to the full length P-INO (pKLP7), and it did not modify the staining pattern in the ovule, as nine of ten transformants showed the *INO*-like pattern of expression. However, in contrast to the effects of the 35S enhancer on all other P-INO element combinations, expression outside the ovule was absent in all ten transformants of pKLP7 (Figure 1Q and 5). This indicates that the full length *INO* promoter can inhibit the activity of the 35S enhancer in regions outside of the ovule. Because this was the longest promoter fragment yet tested it was possible that the failure of the 35S enhancer to produce expression outside of ovules was due to the distance from the minimal promoter. However when we tested the 35S enhancer on a fragment in which POS9 was replaced with a spacer of similar length and G/C content between POSX and POSY (pKLP115, 35S:POSXspacerPOSY::GUS) it showed a similar activity to the POSXPOSY constructs with three of twelve transformants staining in the ovule and five of seven transformants staining in non-ovule regions (Figure 5). Thus, the intact P-INO appears to have a unique activity that can repress activation outside of ovules, and no subregion or smaller combination of subregions of P-INO is able to replicate this activity.

### Mutation of specific sequence motifs in the POS9 region of P-INO

**Figure 6:**
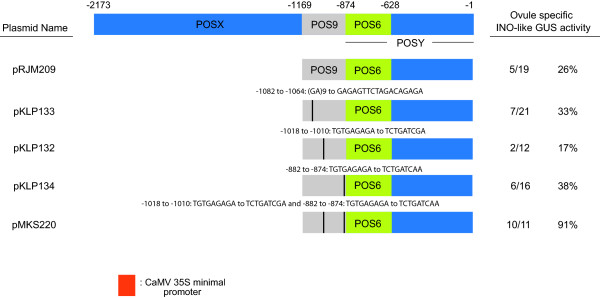
**Effects of mutations in POS9 on promoter activity.** Diagrams of the entire *INO* promoter, promoter deletion and four site-directed mutagenesis constructs are shown. Positions in the diagrams represent the number of base pairs upstream of the putative translational start codon of INO. Ovule specific *INO*-like GUS activity indicates the number of independent plants exhibiting the outer integument specific GUS staining pattern shown by the full-length P-INO relative to the total number of transformants evaluated, and percentage calculated from these values. The frequency of *INO*-like expression for pRJM209 was previously described [12], and was similar to results obtained in the experimental repetition reported here.

Prior [12] and current results showed that the POS9A subregion positively regulates the level of reporter gene expression, whereas the POS9B and POS9C subregions were necessary for producing reporter gene expression within the ovule. The identification of the Basic PentaCysteine (BPC) proteins that bind GA repeats, or possibly purine-rich sequences, and the presence of multiple GA repeats within the POS9 region suggest a potential binding of the BPC proteins to affect P-INO expression [12,25]. In addition, the BPC gene family has been shown to act in a partially redundant manner to contribute to growth of the outer integument of the ovule [26]. Therefore, the specific contribution of the (GA)_9_ repeat within the POS9A subelement was investigated using site-directed mutagenesis to alter the (GA)_9_ repeat at position −1064 to GAGAGTTCTAGACAGAGA. The mutagenesis of the (GA)_9_ repeat did not change the overall pattern or level of expression in a vector containing the altered POS9 in combination with POS6 (pKLP133), as seven out of twenty-one transformants showed the correct pattern and level of expression of the GUS reporter gene (Figure 6). This is similar to the five of nineteen transformants observed with this expression pattern for pRJM209 (POS9POSY::GUS) with the unaltered POS9 region. The GA repeats in this region are conserved, although with some variation in the number of GA repeats, in the *Brassica* species examined (Additional file 1).

A unique nonomer, TGTGAGAGA, is present in both POS9B and POS9C at locations wherein deletions appeared to affect the function of the promoter [12]. A possible role of this element was investigated using site-directed mutagenesis. In the construct containing POS9POSY (pRJM209), the TGTGAGAGA element in POS9C was mutated to TCTGATCAA (pKLP134) but this change did not alter the activity of the reporter gene, as six of sixteen transformants showed GUS staining in the ovules in an INO-like pattern (Figure 6) as compared to five of nineteen in the unmutagenized plasmid. The TGTGAGAGA element in POS9B was mutated to TCTGATCGA, and the resulting pKLP132 also did not abolish activity, as two of twelve transformants showed GUS staining in the ovules in an INO-like pattern (Figure 6). When both mutations were combined into a single construct in pMKS220 we observed a higher frequency of INO-like expression than in the other tested constructs, as ten of eleven transformants showed GUS staining in the ovule (Figure 6). Thus, this motif is not essential for expression, but an activity in suppressing expression cannot be ruled out. This motif is conserved in the *B*. *oleracea* and one of the *B*. *rapa INO* promoter sequences.

## Discussion

The tight spatial and temporal confinement of *INO* gene expression to only one layer of the outer integument [7,11] provides an exemplary model for studies of promoter elements necessary for developmental gene regulation. Previous work indicated that multiple independent regions of the *INO* promoter act in a partially redundant manner and defined the limits of one element (POS9) that included sufficient information for driving specific expression [12]. Herein we delineate a second element with properties like those of POS9, show that conservation of sequence in divergent species is concentrated in these two elements, and show that both positive and negative activities contribute to establishing the *INO* expression domain.

### Subregions of POS9 reconstitute promoter activity but necessary sequence motifs could not be defined

The previously identified POS9 element of P-INO contains regulatory elements sufficient to direct the specific pattern of *INO* expression when combined with extra copies of itself or with either POSX or POSY [12]. The POS9A subregion was shown to enhance the level of reporter gene expression and is less well conserved than the more critical POS9B and POS9C regions that are necessary for reporter gene expression. Our current finding that the tetramerized POS9BC region activated expression of the reporter gene in a similar pattern and level to the tetramerized full-length POS9 provides additional evidence that the POS9A region is not necessary to provide the specific pattern of expression, but rather may be functioning as an enhancer of expression. BPC proteins were identified previously to bind the POS9 region of P-INO in a yeast one hybrid assay, and to preferentially bind GA repeat or purine-rich sequences [12,25]. We hypothesized that the (GA)_9_ repeat located between POS9A and POS9B may represent a binding site for BPC proteins and may contribute to enhancement of expression by the POS9A subregion that includes a (GA)_6_ repeat. Specific mutagenesis of the (GA)_9_ repeat in a POS9POSY::GUS construct did not affect the activity of the reporter gene, suggesting that the (GA)_9_ repeat does not contribute to the enhancing activity of POS9A. It is possible that other GA repeats and purine-rich sequences present in the adjacent POS9A or POS9BC subregions may still permit BPC binding to the *INO* promoter and so the mutagenesis of only one such element may be insufficient to modify the level of activity of the reporter gene. Thus the role of the GA repeats in the regulation of *INO* expression remains unclear.

Previous analyses showed that the POS9B and POS9C subregions were required for normal expression of the reporter gene [12], and identified a unique nonomer sequence, TGTGAGAGA, in both of these subregions. However our results indicate that this unique sequence is not required to maintain the pattern or level of reporter gene expression in our assays. In fact, removal of both such sequences led to the highest frequency of expression among the family of related constructs, indicating a possible role for this sequence motif in negative regulation. The POS9 region shows a high degree of sequence conservation with the *Brassica* species examined, with the lowest level of conservation in the POS9A subregion. Thus, the regulatory elements controlling the specificity of expression would seem to be more highly conserved than an enhancing element. The high degree of overall conservation of sequence present in the region of *Brassica* sequences corresponding to POS9B/C prevents identification of specific regulatory elements necessary for the proper pattern of *INO* gene expression. Within the POS9 region at position −957, we note the presence of a putative binding site for *AINTEGUMENTA* (*ANT*), a transcription factor required for the maintenance of *INO* expression [12]. This site with the sequence of GCACTAGTCTCAACTC shows a good match to the ANT consensus sequence of GCAC(G/A)N(T/A)TCCC(A/G)ANG(T/C) identified by Nole-Wilson and Krizek [27]. However, the ANT protein did not bind to the POS9 region in a yeast one hybrid assay [12]. A putative ANT binding site is also present in the promoters of the YABBY genes *FIL* and *YABBY3* and is adjacent to the binding site of the Kruppel protein that represses gene expression [28]. However, we do not observe a similar Kruppel binding site sequence in the *INO* promoter.

### Functionally redundant elements of P-INO

Prior work showed that POSX or POSY could be added to POS9 to reconstitute the *INO* expression pattern [12]. We now show that a combination of POSX and POSY can also reconstitute this pattern, showing that POS9 is not the exclusive site of tissue-specific information. Further we show that a 246 bp subregion of POSY (POS6), directly adjacent to POS9, also contains regulatory information sufficient, when tetramerized, to promote the proper pattern of reporter gene expression. Thus, two regions of the *INO* promoter contain regulatory sequences sufficient to promote the specific pattern of *INO* expression in the ovule, and both require additional sequences to generate a normal level of expression. Within the POS6 region, deletion analyses suggest the presence of a silencing or negative regulatory element between positions −702 and −756, as the removal of 74 bp from the 3′-terminus of POS6 abolished GUS activity, but this activity was restored when an additional 54 bp was deleted, suggesting that the restored reporter gene activity could be the result of a deletion of sequence bound by a repressor. In analyses of the *FIL* promoter, an abaxial leaf-expressed YABBY gene of Arabidopsis, Watanabe and Okada [5] identified a cis-regulatory element that suppresses the expression of the reporter gene on the adaxial side of the leaf, thereby giving a specific abaxial pattern of expression. However, the putative repressive domain we have identified in the POS6 region of *INO* promoter eliminates all expression from the ovule, and is not just responsible for the polarity of expression. The ectopic expression in the anthers in constructs containing multiple copies of the POS6 region, but not when POS6 is present with other P-INO regions, suggest that the anther expression is repressed by P-INO sequences flanking POS6. Thus, both positive and negative regulatory elements reside within POS6 and other parts of P-INO including POS9 and POSX, and the combination of these elements establishes the ovule-specific pattern of expression.

Both POS9 and POS6 require multiple copies or addition of another element for significant expression from their included tissue-specific regulatory information. For POS6 it appears that the addition of general enhancer activity is sufficient to promote ovule expression since the addition of the 35S enhancer led to lines with ovule expression in the expected pattern. Further, a multimer of the POS6 element is sufficient to promote reporter gene expression in the ovule at a level similar to the full-length promoter or the POS9POS6 elements together. This contrasts with POS9, and all tested combinations of more than one element, where the 35S enhancer had no effect on ovule expression. Further, multiple copies of the POS9 element did not reconstitute the normal level of expression because detection of reporter activity required an elevated substrate concentration. This indicates that the position-specific elements that activate gene expression in POSY are sufficient to promote expression within the ovule but require additional non-specific enhancing elements to activate reporter gene expression to detectable levels whereas POSX and POS9 require additional position-specific enhancers to promote expression.

The ability of the 35S general enhancing element to promote non-ovule expression when in combination with other regions of the *INO* promoter was repressed by the full length P-INO, but not by any less complete combination of sub-regions, even when a spacer sequence was introduced to maintain overall sequence length. Thus, only the full *INO* promoter can act as an enhancer-blocking insulator of the 35S general enhancer. Relatively few examples of sequences with enhancer-blocking activity have been described in plants (reviewed in [29]). The repressive activity is distributed over all three identified subregions of P-INO. This demonstrates that the highly specific expression pattern of *INO* is in part due to active suppression of expression outside of ovules. The POSY region appears to include less of the repressive activity because we observe specific anther expression when multiple copies of POS6 are present. In addition, the POSY region shows a greater response to the presence of the 35S general enhancer than do the other P-INO regions.

### The *INO* minimal promoter region

In addition to the POS6 regulatory element, the POSY region includes another functional domain, the *INO* minimal promoter region. We found that the region including base pairs −509 to +1 could efficiently support expression driven by the 35S enhancer, but that a shorter region beginning at −280 could not. This *INO* minimal promoter does not appear to have a tissue-specific activity, as we did not observe expression in the ovules when the minimal promoter fragment is fused to the 35S enhancer, rather the pattern of reporter gene expression was more similar to the activity of the 35S enhancer with the 35S minimal promoter. We note that the *INO* minimal promoter is large relative to other well-characterized minimal promoters such of those of CaMV 35S (60 bp, [22]) and of a ribulosebisphosphate carboxylase gene (33 bp, [24]).

### Sequence conservation of *INO* promoter elements

Analysis of promoter regions of orthologs of *INO* in *B*. *oleracea* and *B*. *rapa* showed that the POS9, POS6 and minimal promoter regions show a high degree of sequence conservation in *Brassicaceae*. We note a decrease in the degree of sequence similarity in the regions adjacent to the POS9 and POS6 junction, but the sequences at the junction of these two elements are highly conserved, consistent with our analysis showing that deletions at this border significantly disrupted the activity of both elements. The degree of similarity is also less in the POS9A region relative to the POS9B and C regions, consistent with the less essential activity of POS9A. An additional small region of sequence similarity in the POSX region of the *INO* promoter, from position −1711 to −1657, also shows sequence conservation in *Brassica* species. This may be a significant component of the POSX element, but we have not investigated the specific contribution of this region to *INO* expression. The *INO* promoter sequences from more distantly related species, *Carica papaya* and *Vitis vinifera*, were also examined and sequence conservation was not observed (data not shown). Further, the *INO* promoter sequence from the closely related *Arabidopsis lyrata* was compared to *A*. *thaliana* and sequence similarity along the entire length of the promoter did not allow us to distinguish regions of the promoter where conservation of sequence may indicate functional activity (data not shown). The *INO* promoter from *B*. *oleracea* was shown to contain all regulatory information sufficient to promote expression in an AtINO-like pattern in Arabidopsis, as P-BoINO::AtINO:GFP was expressed in the outer layer on the gynobasal side of the outer integument. A construct utilizing this promoter also fully complemented the *ino1* mutant phenotype, thereby demonstrating that the regulatory elements necessary for both the specific expression on the gynobasal side of the outer integument and for the autoregulatory action of the AtINO protein to upregulate and maintain expression driven by P-INO promoter [11] are conserved in the *B*. *oleracea INO* promoter.

A region at the 3′-end of P-INO between −317 and −173 also shows a high level of sequence conservation and our analyses indicate this region may be a necessary part of the *INO* minimal promoter region for basic expression. Part of this region would be deleted in the shortest promoter fragment (deletion to −280) that failed to efficiently function, but was included in the longer fragment (deletion to −509) that did function in conjunction with the 35S enhancer.

## Conclusion

We find that the ovule specific expression produced by the *INO* promoter in the outermost cell layer on the gynobasal side of the outer integument is a result of a combination of position-specific and general enhancers to promote ovule expression, and repressive sequences to prevent expression outside of the ovule. Both types of sequences reside in all three elements we have defined. Positive activity is demonstrated because only combinations of at least two elements (that can be identical elements) produce significant expression. Distributed negative activity is shown by the requirement for all three elements to block general expression driven by the 35S enhancer, and by the ability of POSX and POS9 to block ectopic anther expression driven by POSY. Further, a region of negative activity was found during the deletion analysis of POSY. POSY differs from the other elements in two ways; it was the only element facilitated by the 35S enhancer, and it appears to have weaker activity in blocking expression outside of ovules. Despite the functional similarities between the three identified regions, no common sequence motifs were identified. It is possible that different sequences with redundant activities reside in the elements, or alternatively, common elements may be sufficiently variable to prevent identification. Additional refinement of the location of functional regions, and studies on factors bound to the regulatory elements, or on modification of the DNA or chromatin in these regions may allow resolution of these possibilities.

## Methods

### Plant material

Arabidopsis Landsberg *erecta* (Ler) plants were grown under long day conditions as previously described [30]. Plants were transformed by the floral dip method [31], and transformants were selected by spraying with 6 X 10^-3^% glufosinate ammonium herbicide.

### Promoter-reporter gene constructs

The constructs pRJM77, pRJM209, pRJM307, pRJM293, pCGN23, pRJM199, pRJM106, pRJM160 and pCGN09 have been previously described [11,12].

Deletions from the 5′ terminus of the POSY element of the *INO* promoter were produced using oligonucleotide mutagenesis [21] on pRJM209 [12] to generate eight promoter fragments. Primers P55^′^IIDEL1, P55^′^IIDEL2, P55^′^IIDEL3, P55^′^IIDEL4, POS45^′^DEL1, POS45^′^DEL2, POS45^′^DEL3 and POS45^′^DEL4 (primer sequences used in this study are listed in Additional file 2) were used to generate promoter fragments that were subsequently cloned into the HindIII/NcoI sites of pRJM137 (P-INO::GUS with P-INO replaced in these clonings; [12]) to create pLAW138, pLAW139, pLAW140, pLAW141, pLAW64, pLAW65, pLAW66 and pLAW67, respectively.

Deletions from the 3^′^ terminus of the POSY element of the *INO* promoter were produced by PCR amplification of promoter fragments using pRJM137 [12] as a template with the forward primer 5^′^POS8 with the reverse primers POS43^′^DEL1, POS43^′^DEL2, POS43′DEL3, POS43^′^DEL4 and POS43^′^DEL5. These fragments were cloned into HindII/BamHI sites with the 35S minimal promoter and GUS coding region into pMON999 to create pLAW01, pLAW02, pLAW03, pLAW04 and pLAW05, respectively.

The POS9BC element of the *INO* promoter was amplified from pLMK134 [12] using the primers NEWPOS8for and NEWPOS8rev and cloned into pLITMUS28 (New England Biolabs) to create pLAW117. The tetramer (4XPOS9BC) was created by repeatedly ligating the BglII/BamHI fragment of pLAW117 into BamHI digested pLAW117 to create pLAW120 as previously described for the POS9 element [12]. The 4XPOS9BC element was combined with the 35S minimal promoter from pLAW105 and the GUS coding region from pCGN43 and inserted into pMON999 [11] as a PstI/StuI fragment adjacent to the nopaline synthase polyadenylation region.

PCR was used for site-specific mutagenesis of the POS9 sequence of the *INO* promoter using pRJM209 [12] as a template. Mutations in the (GA)_9_ sequence of the POS9A regions were created using the primers GA9-5^′^ and GA9-3^′^. Mutations in a conserved sequence in the POS9B and POS9C regions were created using the primers POS9Bfor and POS9Brev, and POS9TOPfor and POS9BOTTOMrev, respectively. These PCR products were ligated into the HindIII/NcoI sites of pRJM209 to create pKLP133, pKLP132 and pKLP134, respectively. To create mutations in the conserved sequence of POS9B and POS9C, the POS9TOPfor and POS9BOTTOMrev primers were used with POS9B mutated pKLP132 template.

Deletion of the POS9 sequence from the *INO* promoter was produced by oligonucleotide mediated mutagenesis [21] on pRJM77 with the POS9DEL primer to create pLAW156. The HindIII/NcoI fragment of pLAW156 was cloned into pRJM77 to create pLAW158.

A sequence to act as a spacer between the POSX and POSY regions, in place of POS9 in the *INO* promoter was produced by PCR using the primers BglIIspacer5^′^ and BglIIspacer3^′^ on the *ROC3* gene of Arabidopsis using pCG23 as a template [32] and inserted into pCR4-TOPO (Invitrogen) to make pKLP114. The BglII fragment of pKLP114 was then transferred to pLAW158 and pKLP4 to create pKLP116 and pKLP115, respectively.

The POS6 element of the *INO* promoter was amplified from pLAW158 using the primers POS6Bgl and POS6BamHI and cloned into the BglII and BamHI sites of pBluescript KS- which had been modified to have a BglII site between the EcoRI and BamHI sites to create pKLP57. The BamHI/SacI fragment of pRJM293 (including the 35SMP::GUS combination, [12]) was cloned to pKLP57 to create pKLP60, and the BglII/SacI fragment of pKLP60 was recloned to pRJM293 to create pKLP61. The tetramer of the POS6 element were produced by repeatedly cloning the BglII/BamHI fragment of pKLP57 into the BglII site of pKLP61 to create pKLP63.

The 35S enhancer sequence [22] was amplified using the primers 35S-5^′^Hind and 35S-3^′^SalHind and cloned into the HindIII site of pUC118 to create pKLP2. The HindIII fragment of pKLP2 was then cloned to the HindIII sites in pRJM77, pLAW158, pRJM209, pRJM307, pCGN23, pRJM199, pRJM106, pRJM160 and pCGN09 ([12] and see above) to create pKLP7, pKLP4, pKLP83, pKLP10, pKLP6, pKLP8, pKLP5, pKLP9 and pKLP26, respectively. The correct orientation of the 35S enhancer in these clones was confirmed by digestion at an asymmetric MspI site.

A fragment of the *B*. *oleracea INO* (BoINO) gene was amplified from *B*. *oleracea* using primers based on the Arabidopsis sequence (B-INO1, B-INO2 and B-INO3). The sequence of the BoINO fragment was used to design primers (B-INO4 and B-INO5) that were used to identify a bacterial artificial chromosome (BAC) clone containing this gene from a *B*. *oleracea* library (a gift of Carlos Quiros, UC Davis). A BglII/HindIII fragment of this BAC that included all of the 5^′^-flanking region of the BoINO gene was subcloned into these same sites in pLITMUS28 to create pRB9 (the sequence of the utilized part of the insert of this clone was deposited in GenBank [GenBank:JX682714]). The entire promoter 5^′^-flanking region (from immediately flanking gene to start codon) was amplified with primers PB-INO1 and PB-INO2, adding HindIII and BamHI sites, respectively, and inserted at these sites into pBluescript KS+ to form pRB26. This 5^′^-flanking fragment was combined with the AtINO cDNA into pMON999 to form pLAW181 (P-BoINO::AtINO). The 5^′^-flanking fragment was also combined with an INO cDNA from which the stop codon had been removed and the eGFP coding region [33] to create pRB29 (P-BoINO::AtINO:GFP).

All promoter constructs were shuttled as NotI fragments into the pMLBART [34] binary vector and pMLBART derivatives were transferred to either *Agrobacterium* strain ASE [35] or GV3101::pMP90 [36].

### Microscopy

Tissue was histochemically stained for GUS activity and visualized using differential interference contrast (DIC) light microscopy, and GFP fluorescence was observed as previously described [11].

### Sequence analysis

The *Brassica rapa INO* gene sequences, Bra024599 and Bra012373, were identified through sequence similarity searches of the genetics and genomics databases for Brassica [37]. Sequence comparisons between the Arabidopsis and *Brassica INO* promoters were made using EARS, FSA and ClustalW [18-20].

## Competing interests

The authors declare that they have no competing interests.

## Authors’ contributions

All authors participated in the design of the study; MS, LW, KP and RB performed the experiments. MS, LW and CG did the majority of writing of the manuscript, which was further edited by other authors. All authors read and approved the final manuscript.

## Supplementary Material

Additional file 1 **Alignment of conserved regions of the INO promoters from Arabidopsis thaliana (AtINO), Brassica oleracea (BoINO) and Brassica rapa (BrINO1 and BrINO2) orthologs using the FSA procedures **[20]**.** Click here for file

Additional file 2 Primers used in this study. Click here for file
