# Supplementary material for: Positive- and negative-acting regulatory elements contribute to the tissue-specific expression of INNER NO OUTER, a YABBY-type transcription factor gene in Arabidopsis
Source: BMC Plant Biol. 2012 Nov 13;12:214. doi: 10.1186/1471-2229-12-214 (PMC3583067; doi:10.1186/1471-2229-12-214)
Supplement: Additional file 1 — Alignment of conserved regions of the INO promoters from Arabidopsis thaliana (AtINO), Brassica oleracea (BoINO) and Brassica rapa (BrINO1 and BrINO2) orthologs using the FSA procedures [20]. [file 1471-2229-12-214-S1.doc]

**Additional file 1**

Alignment of conserved regions of the INO promoters from Arabidopsis thaliana (AtINO), Brassica oleracea (BoINO) and Brassica rapa (BrINO1 and BrINO2) orthologs.

POSX similarity region

AtINO CACCAAT-GTCTTGAAGTT--------TTGTTGTTGTTGGCTCTGAT---AACTTGT—T -1665

BoINO CACCAAT-GACTTAATATTGCTTTTGC--------GCTGAATCTGAT---CACTTGT—T -2060

BrINO1 CACCAAT-GTCTTAATATTGCTGTTGC--------GCTGAATCTGAT---CATTTGT—T -1912

BrINO2 CACCAATTGTGTTAATCTTGCTGTTGC--------TCTGAATCTTATCTC-ACTTGTTT -2633

******* * ** * ** ** *** ** * **** *

AtINO TGCTAAGAT -1657

BoINO TGCTAAGAT -2052

BrINO1 GGCTAAGAT -1904

BrINO2 TGCTAAGAT -2652

********

POS9 similarity region

AtINO GTAAGTGAG--AGAGAGAGAAAGCAATAAA-GAGAGGGACAGTAGAAA-------TAAC -1119

BoINO GTACGTC--AGA--------GAGCAATAAAGGAGAGGGACAGTGGAAA-------CAAC -1639

BrINO1 -TACGTC--AGA--------GAGCAATAAAGGAGAGGGACAGTGGAAA-------CAAC -1520

BrINO2 GTGAGTCAGAGA--------GAGCAATAAAGGAGAGGGACAGTGGAAATTGGAAACAAC -2061

* ** * ********* ************ **** ***

AtINO ATGACA-CAGAGAGAAA---GCACTGAAACTTGAAGAAAT------------------- -1083

BoINO ATGA--AGAGAGAGA-----GCACTGAAACTTGAGAATGG------------------- -1565

BrINO1 ATGA--AGAGAGAGAGA---GCACTGAAACTTGAGAATGG------------------- -1445

BrINO2 ATGA—GGAGATAGAGAGAGGCACTGAAACTTGAATTTAGAGATTGAAACTGAGGTGTTT -1952

**** *** *** **************

AtINO --------------------------AGAGAGAGA-GAGAGAGAGAAGA---------- -1061

BoINO --------------------------TT-------TGAGAGAGAGAAAGAGAGAGTGAT -1538

BrINO1 --------------------------TT-------T------------GAGAGAGTGAT -1430

BrINO2 CACAAAAGAAAAAAAGAGATTGAAAC---------TGAGAGAGAGAAGA---------- -1912

AtINO -------GTTTGGGTAAAATGGTCAAGAAA-CAGTTAGCAAAAATGTGAGAGA--AGAA -1012

BoINO AGGAGGACTTTGGGTAAAATGGTCAAGAAAACAGTTAGCAAATTTGTGAGAGAGAA--- -1482

BrINO1 AGGAAGACTTTGGGTAAAATGGTCAAGAAAACAGTTAGCAAATTTGTGAGAGA--A--- -1366

BrINO2 -AGAAGACTTTGGGCAAAATGGTCAAGAAA-CAGTTAGCAAAA-TATGAGAAGGGA--- -1859

****** *************** *********** * *****

AtINO AGACAAAATCCTAAGATTCCGAGTTACTAAAAGTGATGAGTCTCCTAGAAACACCCGCA -953

BoINO GGACAAAATCCTAAGATTCCGAGTTACCAAAAGTGATGAGGCTCCTAGAAACACCCGCA -1421

BrINO1 GGACAAAATCCTAAGATTCCAAGTTACCAAAAGTGATGAGGCTCCTAGAAACACCCGCA -1315

BrINO2 AGACAAAATCCTAAGATTCCGAGTTACCAAAAGTGATGAGTCTCTTAGAAACAACCGCA -1800

******************* ****** ************ *** ******** *****

AtINO CTAGTCTCAACTCACTGCACTTTCATTTTCAGTCTCACTTCA-ACCAAAACTCCAAAAG -895

BoINO CTAGTCCCATCTCATTGCACTTTCACTCTCAATCTCATTCCAAACCTAAACTCCAAA-G -1365

BrINO1 CTACTCTCATCTCATTGCACTTTCACTCTCAATCTCATTCCAAACCTAAACTCCAAA-G -1259

BrINO2 CTAGTCTCAACTCATTGCACTTTCACTCTCAATCTCATTTCAAACCTAAATTCCAAA-A -1742

*** ** ** **** ********** * *** ***** * ** *** *** ******

AtINO ACTGT--TGAGTTTTGTGAGAGA -874

BoINO ACTGTATTTTG-TTTGTGAGAGA -1344

BrINO1 ACTGTATTGTG-TTTGTGAGAGA -1238

BrINO2 ACTGCATTGTGTTTTGTGGTTGG -1720

**** * * ****** *

POS6 similarity region

AtINO TTCATTAAATCCCATTTAAAGAGACAACTACACATGAGACAAAGAGGCATTTCTTTCTC -816

BoINO TTCAATAAATCCCATTTAAAGAGACAACTACACATGAGACAAAGAGGTATTTTT-TCTC -1286

BrINO1 CTTAATAAATCCCATTTAAAGAGACAACTACACATGAGACAAAGAGGTATTTTTTTCTC -1179

BrINO2 TTCAATAAATCCCATTTAAAGAGACAACTACACATGAGACAAAGAGGAATTTCTCTCTC -1661

* * ****************************************** **** * ****

AtINO AACCTCACTTGTTTTTTTCTTTCTTTCTAACTTGCAAACCAAACCACACCA-CCACATC -758

BoINO TATTTCATTTGTTTT----TTTCTTTCTAACTTGCAAACCAATCCAAAA--------CC -1239

BrINO1 AATTTCATTTGTTTT----TTTCTTTCTAACTTGCAAACCAATCCAAAC-ACACAC-CC -1126

BrINO2 AACTTCATTTGTCTTTTTTTCTCTTTCTAACTTGCAAACCAATCTACTACAC--ACACC -1604

* *** **** ** * ********************* * * *

AtINO ACATCATTTATTAACCCCAGCTCTCTTCGAT-GTCTATCTATCTCCT------CTTGTG -706

BoINO ACATCATTTATTA-CCTATCCTCTCTTCTATAGACTGTCTATCTGCTCTTGTGCTTG-G -1182

BrINO1 ACATCATTTATTA-CCTATCCTCTCTTCTATAGACTGTCTGCCTGCTCTTGCGCTTGTG -1068

BrINO2 ACCTCATTTATTA-CATTTCCTCTCTTTGATCTAATA-CTATC------------TGTG -1559

** ********** * ******* ** * ** * *

AtINO CA----ACGTGCCTTTCCAT--TT--TCACATGTTCATTTTT--AATACTCATACTCTT -657

BoINO CAACGTACGTGGCTTTCGTTTTTTTTTTACATGTTCATCATAACAATCTACATACTCTA -1123

BrINO1 CAACGTACGTGGCTTCAATATTTT--TTCCATGTTCATCGTAACAATCTACATACTCTA -1011

BrINO2 CA----ACGTGGCTTTCCAT--TT--TGACATGTTCATTGTAACACCGTACATAATCTT -1508

** ***** *** ** * ********* * * **** ***

AtINO TACCATCGA-------------------TTTTTATCTCTTGTTATTTT -628

BoINO CACCATCAGA----------TA------TATATACATTTTATTATTCT

BrINO1 CACCATGAGT--------TGTA------TATATACATTTTGTTATTCC

BrINO2 CACCAATGGTCATAGATTTATATATATT-----AGCTTTTGTTATTCT

**** * * ** *****

INO POSY MP similarity region

AtINO TTTATTTTATTTTAGAGAAGAAAATGTAGTTTGAATATTAAGGAGTTAGGAATGTGATT -262

BoINO TTTATTTTCTTTTA-AAAAGAAAATGTAGTTT----ACTAAGGAGTTAGGAATGTGATT -183

BrINO1 TTTATTTTCTTTTA-AAAAGAAAATGTAGTTT----ACTAGGGAGTTAGGAATGTGATT -523

BrINO2 TTTATTTTCTTTAG-AAAAGAAAATGT----------------AGTTAGGAATGTGATT -313

******** *** * ********** ****************

AtINO AATAGAAAGAATGAGAATCATTTACTGCTTACAGCTCATAGAGACTATGAAGATTCCAA -203

BoINO AATAGTGAGAATGAGAATCATTTACTACTCACAGCTCATCGAGACGATGAAGATTCCAA -124

BrINO1 AATAGTGAGAATGAGAATCATTTACTACTCACAGCTCATCGAGACGATGAAGATTCCAA -464

BrINO2 AATAGAAAGAATGAGAATCATTTACTACTCACAGCTCATCGAAACGATGAAGATTCCAA -254

***** ******************* ** ********* ** ** *************

AtINO TTATTACAA-GTGAGATAGAGACGTACATAA -173

BoINO TTATTAGAA-GTGGGATAGAGACGCACATAA -94

BrINO1 TTATTAGAA-GTGGGATAGAGACGCACATAA -434

BrINO2 TTATTAGAAAATGAGATGGAGACGTACATAA -223

****** ** ** *** ****** ******
