# Supplementary material for: Positive- and negative-acting regulatory elements contribute to the tissue-specific expression of INNER NO OUTER, a YABBY-type transcription factor gene in Arabidopsis
Source: BMC Plant Biol. 2012 Nov 13;12:214. doi: 10.1186/1471-2229-12-214 (PMC3583067; doi:10.1186/1471-2229-12-214)
Supplement: Additional file 2 — Primers used in this study. [file 1471-2229-12-214-S2.docx]

**Additional file 2**

Primers used in this study

P55’IIDEL1 (AAAGACTGTTGAGTTTTGTGAGAGATCTAATCCCATTTAAAGAGACAACT)

P55’IIDEL2 (AAAGACTGTTGAGTTTTGTGAGAGATCTCTACATGAGACAAAGAGGCA)

P55’IIDEL3 (AAAGACTGTTGAGTTTTGTGAGAGATCTCATTTCTTTCTCAACCTCACTT)

P55’IIDEL4 (AAAGACTGTTGAGTTTTGTGAGAGATCTTTGTTTTTTTCTTTCTTTCTAA)

POS45’DEL1 (AAAGACTGTTGAGTTTTGTGAGAGATCTCCAAACCACACCACCACCATCAC)

POS45’DEL2 (AAAGACTGTTGAGTTTTGTGAGAGATCTCTATCTCCTCTTGTGCAACGTG)

POS45’DEL3 (AAAGACTGTTGAGTTTTGTGAGAGATCTGATTTTTATCTCTTGTTATTTT)

POS45’DEL4 (AAAGACTGTTGAGTTTTGTGAGAGATCTTCTTGTATGCTTATTTATCAG)

5’POS8 (CCCAAGCTTCACAAAATAAGGAGATGAGAG)

POS43’DEL1 (CGCGGATCCCTGATAAATAAGCATACAAGAT)

POS43’DEL2 (CGCGGATCCAAAATAACAAGAGATAAAAATCG)

POS43’DEL3 (CGCGGATCCGTTGCACAAGAGGAGATAG)

POS43’DEL4 (CGCGGATCCGTGATGTGGTGGTGTGGT)

POS43’DEL5 (CGCGGATCCTGCAAGTTAGAAAGAAAGAAAA)

NEWPOS8for (AAAACTGCAGAGATCTGTAAAATGGTCAAGAAACAGT)

NEWPOS8rev (GCGGGATCCCTCACAAAACTCAACAGTCT)

POS6Bgl (CCAGATCTTCATTAAATCCCATT)

POS6BamHI (TTGGATCCAAAATAACAAGAGAT)

POS9Bfor (AATCTGATCGAAGAAAGACAAAATCCT)

POS9Brev (TTCGATCAGATTTTTGCTAACTGTTTC)

POS9TOPfor (TTTTCTGATCAATTCATTAAATCCCAT)

POS9BOTOMrev (TGAATTGATCAGAAAACTCAACAGTCT)

GA9-5’ (GAGTTCTAGACAGAGAAGAGTTTGGG)

GA9-3’ (TCTGTCTAGAACTCTCTATTTCTTCA)

35S-5’Hind (CTCGAAGCTTGGTCCGATGTGAGACTTTTCAA)

35S-3’SalHind (CTCGGTCGACAAGCTTATCACATCAATCCACTTGCTTT)

BglIIspacer5’ (CATAGATCTCTCAACAACATTCAATCCCTCAA)

BglIIspacer3’ (CATAGATCTATTACACTACAAAGGATCAAGCTT)

B-INO1 (TACCGTTTACAAGCTTGTCAATG)

B-INO2 (TGAAAGCTTCCTTGTGAGCCAT)

B-INO3 (TGTAAGCTGAAGGAGCTCTTTG)

B-INO-4 (GTGGGCACTGCACAAGCCTTC)

B-INO-5 (TGAGCCATGCTTGGATTCTGAG)

PB-INO1 (ATTAGAAGCTTGTGTTCTGTTTTTTTCTTTCTTGC)

PB-INO2 (GCCATGGATCCTTTTTGTCATAGAGAGAGAAAG)
